# Outcomes of Recurrent Nasopharyngeal Carcinoma Patients Treated With Salvage Surgery: A Meta-Analysis

**DOI:** 10.3389/fonc.2021.720418

**Published:** 2021-10-08

**Authors:** Yekai Feng, Zhimei Dai, Ruicheng Yan, Feng Li, Xiaosheng Zhong, Haoxin Ye, Caiqing Chen, Shaochong Fan, Cheng Qing, Yong Pan, Haiying Sun

**Affiliations:** ^1^ Department of Otolaryngology–Head and Neck Surgery, The Forth Affiliated Hospital of Guangzhou Medical University, Guangzhou, China; ^2^ Department of Otolaryngology–Head and Neck Surgery, Affiliated Hospital of Guangdong Medical University, Zhanjiang, China; ^3^ Department of Otorhinolaryngology, Union Hospital, Tongji Medical College, Huazhong University of Science and Technology, Wuhan, China

**Keywords:** outcome, adjuvant therapy, surgery, recurrent nasopharyngeal carcinoma, meta-analysis

## Abstract

**Objective:**

To assess the efficacy of treatment outcomes of salvage surgery for recurrent nasopharyngeal carcinoma (rNPC).

**Methods:**

We conducted a detailed search of the literatures in biomedical databases published from January 1990 to December 2020. The main research features and results of interest were retrieved from the articles that met the selection criteria for meta-analysis.

**Results:**

A total of 21 articles with 778 patients were included, 17 of which met the meta-analysis inclusion criteria. The pooled 2-year overall survival (OS), 5-year OS, and 2-year disease-free survival (DFS) were 71%, 50% and 61%, respectively. Subgroup analysis was conducted with postoperative adjuvant therapy. The pooled 2-year OS, 5-year OS and 2-year DFS of the postoperative adjuvant therapy group compared with the surgery alone group were 69% vs 72%, 44% vs 56%, and 77% vs 54%, respectively. Univariate and multivariate analyses were performed on 178 patients with detailed individual postoperative survival data in 10 articles. On multivariate analysis, recurrent T (RT) stage and adjuvant therapy were independent predictors of outcomes.

**Conclusions:**

This meta-analysis indicated that recurrent NPC patients can obtain survival benefits from salvage surgery. Accurately assessing the RT stage of the tumor and choosing the appropriate surgical method are important to the success of the surgery. Although the prognostic factors influencing outcome have been studied, conclusive data on the survival benefits are still lacking. Random controlled trials (RCTs) to compare surgery alone and postoperative adjuvant therapy are needed in patients with positive margin status after salvage surgery.

## Introduction

Nasopharyngeal carcinoma (NPC), which originates from nasopharyngeal epithelial cells, is a coon malignant tumor that occurs in the head and neck (1). The primary treatment strategy for NPC is radiotherapy with or without chemotherapy (2). However, approximately 7% to 15% of patients have persistent or recurrent disease after radical radiotherapy, and 10% to 40% of patients experience recurrence within 1 to 2 years after initial treatment (2, 3).

At present, there is still no standardized management strategy for recurrent NPC (rNPC). Surgery is often the first choice for recurrent locoregional NPC. Intensity-modulated radiotherapy (IMRT) can be chosen as a salvage treatment for unresectable disease. Targeted therapy and chemotherapy can be considered for patients who cannot undergo or refuse to receive reirradiation. Palliative chemotherapy is the main choice for patients with distant metastasis (4, 5). Radiotherapy resistance is the main reason of NPC relapse within 1 year and fatal complications caused by irradiation makes the situation more worse (6). It is reasonable that further radiotherapy (RT) or chemotherapy (CHT) might lead to undesirable survival outcomes. The development of salvage surgery provides an alternative treatment.

In this study, we carried out a meta-analysis of the long-term results of patients who underwent surgery with or without adjuvant therapy for recurrent NPC. The combined OS and DFS rates outcomes were reported. At the same time, subgroup analysis of postoperative adjuvant therapy was performed. We also performed univariate and multivariate analyses to identify prognostic factors in a series of patients with detailed postoperative survival data.

## Materials and Methods

### Search Strategy

A systematic search of the PubMed, Embase, Cochrane Library, and Web of Science databases and 2 major Chinese databases, CNKI and Wanfang, were conducted in December 2020. The search strategy was predefined. The following free terms and medical subject headings were included: “nasopharyngeal,” “nasopharyngeal diseases,” “nasopharyngeal neoplasms,” “nasopharyngeal carcinoma,” “recurrence,” “surgery,” and “survival.” We limited the scope of our research to studies that only targeted humans and published in Chinese and English. The publication time was restricted from 1990 to 2020.

### Inclusion and Exclusion Criteria

Studies that met all of the following inclusion criteria were selected: (1) Study population: patients with histologically proven, locally recurrent, nonmetastatic NPC receiving a primary and radical radiotherapy; (2) Treatment modality: salvage surgery for rNPC patients with or without adjuvant therapy; (3) Outcomes: the results of OS rate and DFS rate in patients who treated postoperative adjuvant therapy and surgery alone; (4) Study design: randomized controlled trials, retrospective and prospective cohort, and case series were included. Case reports, repeatedly published data, studies without adequate data and studies without full text were excluded.

### Data Collection and Extraction

Data were extracted by two independent reviewers (Y.F. and Z.D.). The following data were collected from the full text of articles: The characteristics of author, publication language, number of patients, main clinical features of patients, treatment approaches, postoperative adjuvant therapy and survival rate; The Kaplan-Meier survival curve was used in the way introduced by Parmerl et al. (7) to obtain the required survival data when the survival data were not obtained directly from the articles; Data from studies with detailed individual postoperative adjuvant treatment data were extracted separately.

### Assessment of Study Quality

Each study’s quality was assessed by the Methodological Index For Non-randomized Studies (MINORS) (8). There are total of 12 evaluation indicators, each of which is divided into 0 to 2 points. Scoring method: 0 point means not reported; 1 point means reported but insufficient information; 2 point means reported and provided sufficient information. The first 8 items are designed for no-control studies. The last 4 and the first 8 items are designed for studies with the control group. Articles with a score of 0-8 are low-quality, 9-16 are classified as medium quality, and 17-24 are classified as high-quality. Two reviewers scored independently. If the scoring results are inconsistent, it will be determined through discussion or consultation with a third independent senior oncologist, and finally an agreement is reached.

### Statistical Analysis

In this study, we conducted the meta-analysis using software STATA version 15.0 (StataCorp LLC, College Station, TX). The random effects model (9) was adopted when heterogeneity was detected (I^2^ > 50%). Sensitivity analysis, Meta-regression and subgroup analyses were used to explore the source of the heterogeneity among the studies. The univariate and multivariate analysis of 178 patients with detailed survival data was performed by the IBM SPSS Statistics Version 21. OS and DFS were calculated by the Kaplan-Meier method and compared by the log-rank test. A 2-tailed p < 0.05 indicated statistical difference. Factors that achieved significance on univariate were included in the Cox proportional rate hazard model for multivariate analysis to identify independent significant prognostic factors.

## Results

**Figure 1:**
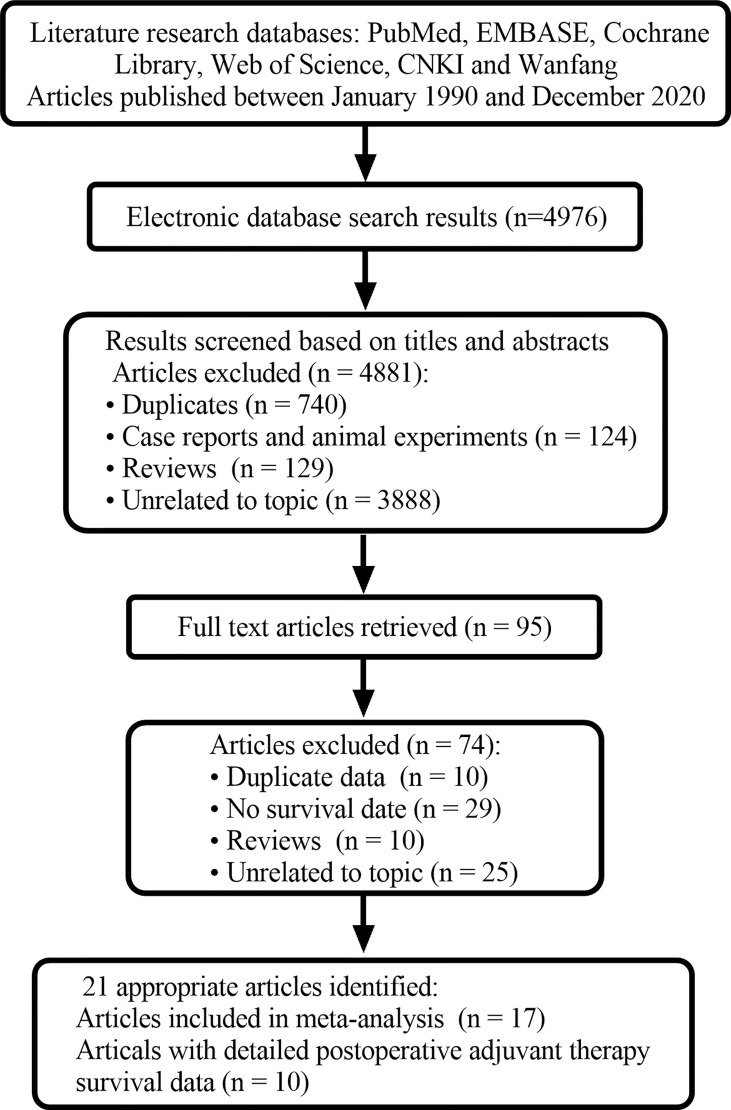
PRISMA flow diagram.

A total of 4976 related publications were retrieved. 4881 articles were excluded because they were duplicates, systematic reviews, animal experiments, case reports, or unrelated to the current analysis. In addition, 95 studies were evaluated later. After reading the full texts, 74 articles were excluded. The main reasons for exclusion are listed in **Figure 1**. Finally, 21 articles were screened out, 17 of which were included in the meta-analysis since they had a sample size of greater than 10 (10–26). Ten articles provided detailed survival data (21–30). The average MINORS score of the included articles was approximately 10 points. There was a medium quality of methodological heterogeneity in this research.

**Figure 2:**
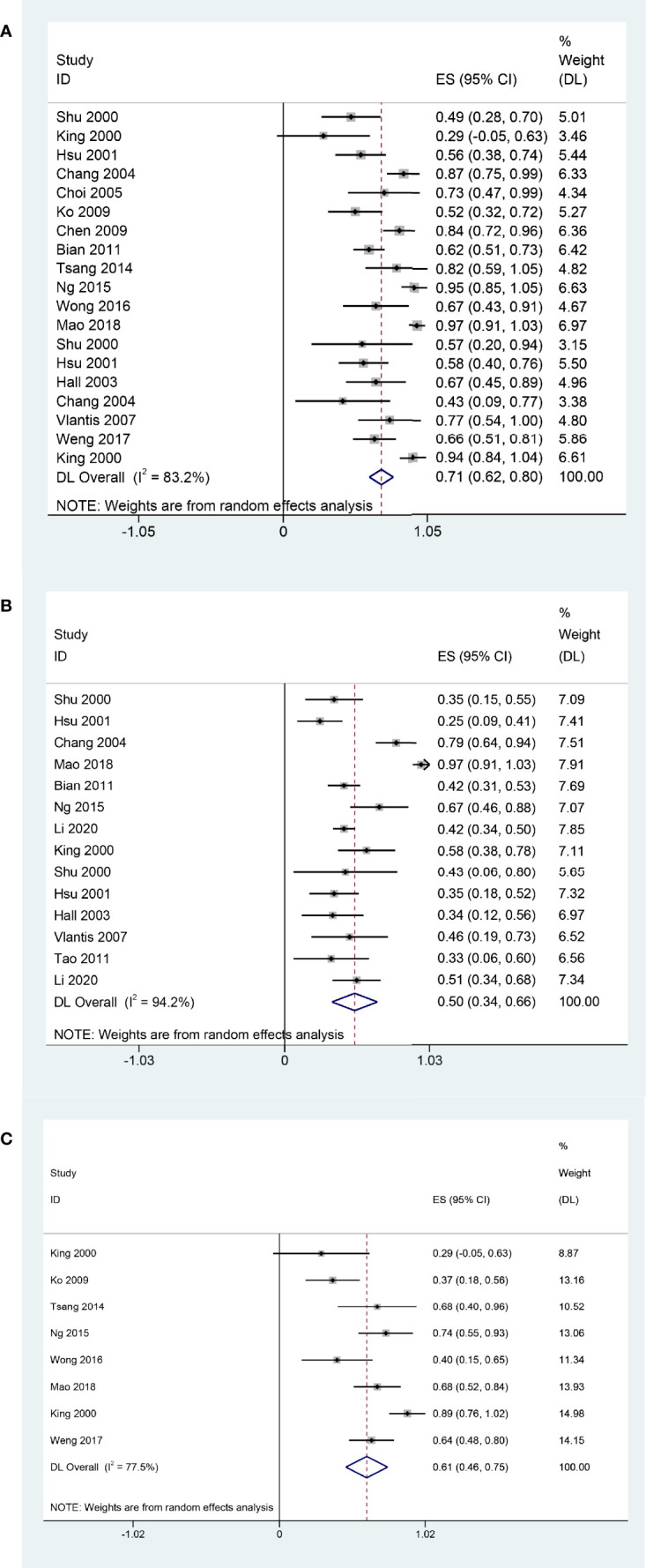
Forest plot of meta-analysis Pooled 2-year OS **(A)**; Pooled 5-year OS **(B)**; Pooled 2-year DFS **(C)**.

The main clinical characteristics are shown in **Table 1**. The pooled 2-year OS, 5-year OS, and 2-year DFS that experienced surgery with or without adjuvant therapy for rNPC were 71% (95% CI, 62%-80%, I^2^ = 83.2%, p < 0.05, **Figure 2A**), 50% (95% CI, 34%-66%, I^2^ = 94%, p < 0.05, **Figure 2B**), and 61% (95% CI, 46%-75%, I^2^ = 77.5%, p < 0.05, **Figure 2C**), respectively. There was high heterogeneity indicated by the I^2^ value being > 50%; thus, the potential causes of heterogeneity and bias were further investigated.

**Table 1:** Main characteristics of the articles included in the meta-analysis.

Author	Published language	Year	M/F (n)	n (rT classification)	Approach		Margins		Adjuvant therapy	Reported outcome of interest			MINORS
					Endoscopic	Open	Positive	Negative or Close		2-year OS (%)	2-year DFS (%)	5-year OS (%)	
Mao et al. (11)	Chinese	2018	21/10	31rT1	31	0	1	30	0	96.6	67.5	96.6	9
Tao et al. (16)	Chinese	2011	23/14	37(-)	8	29	12	0	12			33	10
							0	25	19				
Bian et al. (15)	English	2011	50/21	71(27rT1, 29rT2, 14rT3, 11rT4)	0	71	27	44	0	62.1		42.1	10
Wong et al. (12)	English	2016	9/6	15(2rT3, 13rT4)	15	0	6	9	0	66.7	40		10
Tsang et al. (21)	English	2014	7/5	12(8rT1, 4rT3)	-		0	11	0	81.8	66.7		9
							1	0	1				
Ko et al. (22)	English	2009	21/7	28(12rT1, 16rT2a)	28	0	0	25	0	51.9	37.1		10
							3	0	3				
King et al. (20)	English	2000	28/3	31(20rT1, 9rT2, 2rT3)	0	31	23		23	93.6	89	57.6	11
							7		0	29	29	-	
Shu et al. (26)	English	2000	24/4	28(16rT1, 9rT2, 2rT3, 1rT4)	0	28	0	21	0	49.2		34.5	11
							7	0	7	57.1		42.9	
Chang et al. (24)	English	2004	30/8	38(16rT1, 4rT2, 11rT3, 7rT4)	0	38	0	30	0	86.7		78.8	8
							8	0	8	42.9			
Hsu et al. (19)	English	2001	47/13	60(10rT1, 18rT2, 22rT3, 10rT4)	0	60	32	0	29	58		35	10
							0	28	0	56		25	
Ng et al. (14)	English	2015	14/6	20(18rT1, 2rT2)	0	20	0	20	0	95.3	73.6	66.7	11
Vlantis et al. (18)	English	2007	61/18	79(39rT1, 28rT2, 10rT3, 2rT4)	0	79	0	36	22				10
							0	13 close	13	77		46	
							30	0	25				
Li et al. (10)	English	2020	132/57	189(55rT1, 42rT2, 64rT3, 28rT4)	189	0	32	1	33			51	11
							0	156	0			41.7	
Chen et al. (17)	English	2009	-	37(17rT1, 18rT2, 2rT3)	37	0	1	36	0	84.2			9
Choi and Lee (23)	English	2005	7/4	11(4rT1, 4rT2, 2rT3, 1rT4)	0	11	2	9	0	72.7			9
Hall et al. (25)	English	2003	12/6	18(1rTx, 6rT1, 7rT2, 1rT3, 3rT4)	0	18	-		18	67		33.5	10
Weng et al. (13)	English	2017	26/10	36(8rT1, 9rT2, 8rT3, 11rT4)	36	0	3	33	36	66	64		11

–, not available; DFS, disease-free survival; OS, overall survival; F, female; M, male; MINORS, Methodological Index for Non-Randomized Studies.

Meta-regression analysis showed that rT stage (Tau^2^ = 0.02315; p = *0.209*), postoperative adjuvant therapy (Tau^2^ = 0.0266; p = *0.718*), margin status (Tau^2^ = 0.02377; p = *0.14*), and surgical approach (Tau^2^ = 0.027; p = *0.514*) may not associated with heterogeneity. We further conducted a subgroup analysis of postoperative adjuvant therapy. In this subgroup analysis, we performed exploratory sensitivity analysis to find potential causes of heterogeneity. Sensitivity analysis of the pooled 2-year OS revealed that the postoperative adjuvant treatment outcomes of King et al. (20) might have had an influence on clinical heterogeneity.

**Figure 3:**
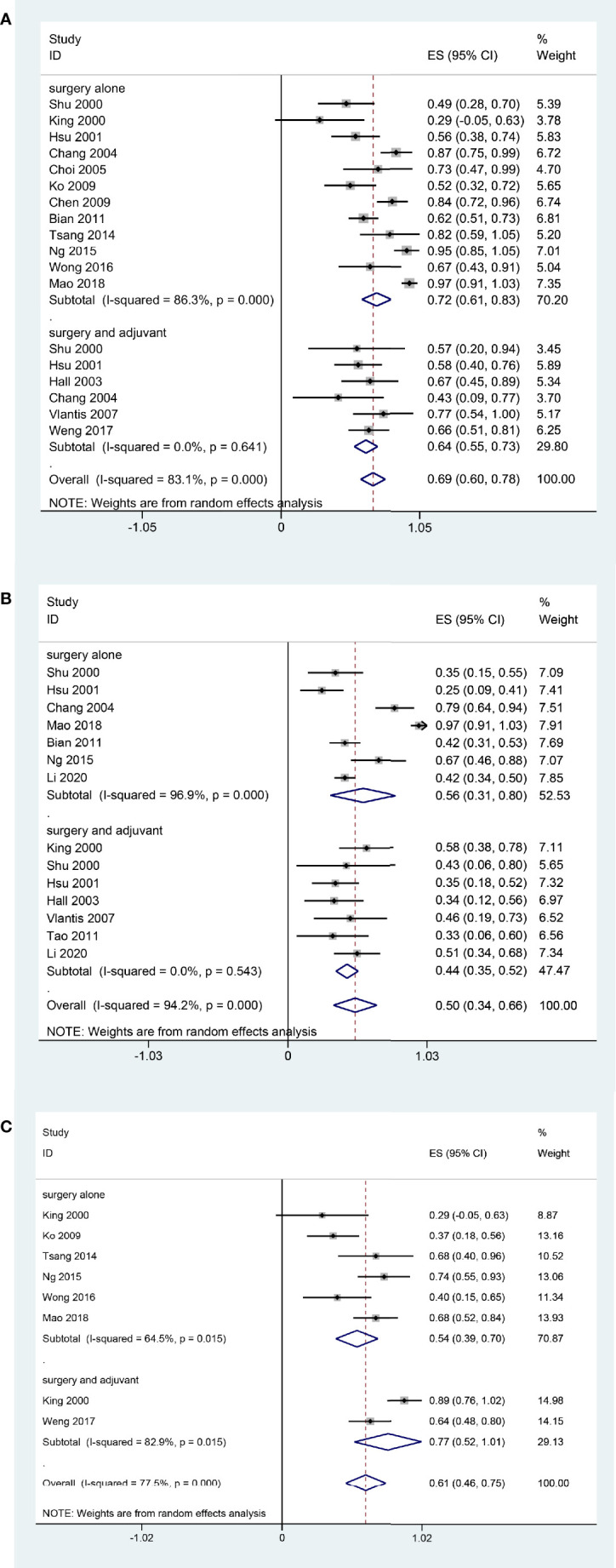
Forest plot of subgroup meta-analysis Pooled 2-year OS **(A)**; Pooled 5-year OS **(B)**; Pooled 2-year DFS **(C)**. Subgroups were stratified according to the postoperative adjuvant therapy status of the patients in each study.

In the subgroup analysis, patients underwent surgery alone had a better 2-year OS rate (72%, 95% CI, 61%-83%, I^2^ = 86.3%, p < 0.05, **Figure 3A**) than those underwent surgery and adjuvant therapy (64%, 95% CI, 55%-73%, I^2^ = 0.00%, p *= 0.641*, **Figure 3A**). The 5-year OS was 44% (95% CI, 35%-52%, I^2^ = 0.00%, p *= 0.543*, **Figure 3B**) in the postoperative adjuvant therapy group and 56% (95% CI, 31%-80%, I^2^ = 96.9%, p < 0.05, **Figure 3B**) in the surgery alone group. The 2-year DFS of the postoperative adjuvant therapy group was 77% (95% CI, 52%-1.01%, I^2^ = 77.5%, p < 0.05, **Figure 3C**), which was higher than that of the surgery alone group (54%, 95% CI, 39%-70%, I^2^ = 64.5%, p < 0.05, **Figure 3C**).

We conducted univariate and multivariate analysis on 178 patients with detailed survival data related to postoperative adjuvant treatment. There were 131 males and 47 females. Their follow-up time was 1-117 months, and the average follow-up time was 26 months. Sixty-six patients underwent RT after surgery, 12 underwent surgery and CHT, and 125 patients underwent surgery alone. The detailed data of each patients are suarized in **Supplemental Table S1**.

There was no significant difference in the distribution of gender, margin status, and recurrent T stage between the open surgery group and the endoscopic surgery group. However, we found there was significant association between surgical approach and adjuvant therapy (p *= 0.010*). In the open surgery group, 62 (65.3%) patients underwent surgery alone, and 33 (34.7%) patients received adjuvant RT after surgery. In the endoscopic surgery group, 63 (75.9%) received surgery alone, 3 (3.6%) received adjuvant RT, 12 (14.4%) received adjuvant CHT, and 5 (6.1%) received postoperative concurrent chemoradiotherapy (CCRT). We further compared the patients who treated with surgery alone, the 5-year OS was 77.0% in the open surgery group and 82.5% in the endoscopic surgery group (*p >* 0.05), the 2-year DFS was 85.0% in the open surgery group and 72.5% in the surgery alone group (*p >* 0.05). In the open surgery group, the 5-year OS was 35.2% in the postoperative RT group and 77.0% in the surgery alone group (*p <* 0.05). The 2-year DFS was 37.3% in the postoperative RT group and 85.0% in the surgery alone group (*p* < 0.05). In the endoscopic surgery group, 12 patients received adjuvant CHT. Compared with the 2-year OS (82.5%) in the surgery alone group, the 2-year OS was 67.3% in the adjuvant CHT group (p <0.05).

**Figure 4:**
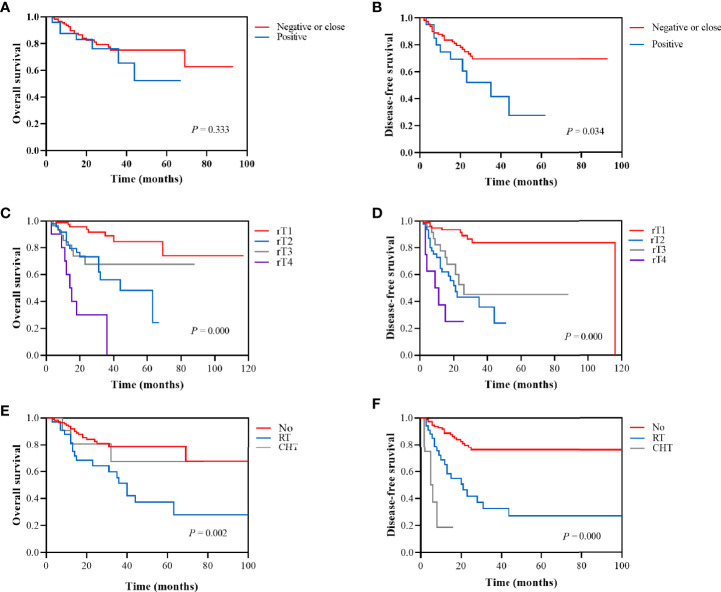
Kaplan-Meier survival analysis according to margin status (negative or close vs positive): **(A)** 2-year OS was 81.0% vs 76.2%. **(B)** 2-year DFS was 73.3% vs 52.0%. Kaplan-Meier survival analysis according to recurrent T stage (rT1, rT2, rT3, and rT4): **(C)** 2-year OS was 93.7%, 73.1%, 67.6%, and 30.0%, respectively. **(D)** 2-year DFS was 93.3%, 43.1%, 52.5%, and 25.0%, respectively. Kaplan-Meier survival analysis according to adjuvant therapy (No, RT, and CHT): **(E)** 2-year OS was 82.6%, 64.1%, and 80.8%, respectively. **(F)** 1-year DFS was 88.7%, 65.3%, and 16.7%, respectively.

The prognostic factors for recurrent NPC are shown in the **Table 2**. Margin status (**Figure 4B**), recurrent T stage (**Figures 4C, D**), adjuvant therapy (**Figures 4E, F**) affected the survival outcomes of patients. The variables considered significant in the univariate were included in the Cox multivariate analyses. Two variables (recurrent T stage and adjuvant therapy) were independent risk factors for the DFS of recurrent NPC in the Cox multivariate analyses (**Table 3**).

**Table 2:** Clinical Characteristics and univariate analysis of prognostic factors.

Variables	No. of patients(%)	P Value	
		DFS	OS
Sex		0.640	0.940
Male	131 (73.6)		
Female	47 (27.7)		
Surgical Approach		0.795	0.097
open	95 (53.4)		
endoscopic	83 (44.6)		
Margin Status		0.034	0.333
negative or close	121 (82.9)		
positive	25 (17.1)		
Recurrent T Stage (4 levels: T1, T2, T3, T4)		0.000	0.000
T1	84 (47.5)		
T2	50 (28.2)		
T3	30 (16.9)		
T4	13 (7.3)		
Adjuvant Therapy		0.000	0.002
(3 levels: not given, RT, CHT)			
No	125 (72.3)		
RT	36 (20,8)		
CHT	12 (6.9)		

OS, overall survival; DFS, dieases free survival; RT, radiotherapy; CHT, chemotherapy.

**Table 3:** Cox multivariate regression analysis of disease free survival at 2 years.

Variables	P Value	HR	95% CI	
			Lower	Upper
Margin Status	0.082	4.169	0.834	20.82437
Recurrent T Stage				
T1	0.000			
T2	0.000	8.832	3.384	23.052
T3	0.003	5.382	1.755	16.508
T4	0.000	12.814	3.099	52.989
Adjuvant Therapy				
No	0.000			
RT	0.033	6.098	1.157	32.138
CHT	0.000	35.744	9.917	128.832

CI, confifence intervals; HR, hazard ratio compared to the first mentioned variable; RT, radiotherapy; CHT, chemoterapy.

## Discussion

Reirradiation, with or without chemotherapy, is a treatment strategy for rNPC. However, it is related with normal tissue injury that results in a rise of mortality and treatment-related morbidity and influences the quality of patients’ life (31, 32). Salvage surgery can achieve a better survival rate with lower treatment-related complications than IMRT or two-dimensional conventional radiotherapy (17, 29, 33). In this study, we aim to assess the efficacy of treatment outcomes in salvage surgery for recurrent nasopharyngeal carcinoma.

In our study, the results of meta-analysis showed that the pooled 2-year OS, 5-year OS and 2-year DFS rates were 71%, 50% and 61%, respectively, indicating that the majority of these patients can obtain survival benefits from surgery, which is comparable to the survival rate of 189 patients reported by Wang et al. (10). In this subgroup analysis, the 2-year OS rate and 5-year OS rate in the surgery alone group were superior to those in the postoperative adjuvant therapy group. The 2-year DFS rate in the postoperative adjuvant therapy group was 77%, which was higher than that the surgery alone group (54%). We further retrieved individual patient data with detailed survival results to compare the survival rate of 178 patients who underwent surgery, and found that recurrent T stage and adjuvant therapy were independent risk factors for the DFS of recurrent NPC in the Cox multivariate analyses.

Studies on the effects of adjuvant therapy on the prognosis of patients have been reported. According to following up 79 patients who were treated with surgery, Vlantis et al. (18) found that the adjuvant radiotherapy may not associate with an additional benefit. That is because the clear margin group, of whom only 61% received postoperative radiotherapy, showed a better survival rate than the positive margin group, of whom 83% received postoperative radiotherapy. You et al. (33) published a case-matched study comparing salvage endoscopic nasopharyngectomy with IMRT for selected local recurrent T1-T3 NPC patients. Their results suggested that the improvement in the OS rate in patients who treated with salvage endoscopic nasopharyngectomy compared with salvage IMRT may be associated with a reduction in the risk of reirradiation injury, rather than the elimination of radiation-resistant disease or a reduction in the risk of local recurrence and distant metastasis. However, A meta-analysis published in 2014 showed that postoperative adjuvant therapy is an effective treatment, with 5-year OS rates of 67% vs 39% in the postoperative adjuvant therapy group compared the surgery alone group (34). King et al. (20) previously described those 31 patients routinely received postoperative radiotherapy and found that nasopharyngectomy supplemented by postoperative radiotherapy achieved significant survival and tumor control in selected recurrent NPC. In our study, there was no significant difference in OS between the clear margin group, of whom only 1.7% received postoperative RT and 6.7% received postoperative CHT, and the positive margin group, of whom 76.2% received postoperative RT and 9.5% received postoperative CHT. Patients with positive margins are recoended to receive RT after surgery. In addition, considering that only two studies were included in the postoperative adjuvant therapy group in the DFS subgroup analysis, there is insufficient data to demonstrate an improved DFS benefit for patients who underwent adjuvant therapy after surgery. Due to the limited number of cases, it is difficult to conduct control studies with large samples, and there is still a lack of convincing evidence-based medicine. We cannot ignore the deviation of highly selected patients.

In the past, advanced tumor invading the internal carotid artery (ICA) and skull base is considered unresectable. With the refinement of imaging, cooperation with ophthalmologist and neurosurgeons, and the development of endoscopic surgery and equipment, more selected advanced tumor including invasion of the ICA can be radically removed (10). However, most surgeons mainly focus on early rNPC, ignoring the research of advanced rNPC. Endoscopic nasopharyngectomy reported by Mao et al. (11) described a 5-year OS rate is 96.6% in 31 early rNPC patients. Liu et al. (35) showed that the 2-year OS rates in rT1, rT2, rT3, rT4 were 82.2%, 47.4%, 70.5%, and 36.8%, respectively. Ng et al. analyzed 20 patients (18 with rT1, 2 with rT2) treated with open surgery, and the results of 2-year OS was 95% (14). Bian et al. (15) showed that the 2-year OS rates for recurrent T1, T2, T3, and T4 disease after open surgery were 79.8%, 66.7%, 42.5%, and 10.6%, respectively. Hao reported the 5-year OS rates in stage I, stage II, stage III, and stage IV disease after open surgery were 64.8%, 38.1%, 25.9%, and 46.9%, respectively (36). In our study, rT3 group is superior to rT2 group, because most patients with rT3 are highly selective patients, and the lesions are confined to paranasal sinus. rT2 tumor confined to the parapharyngeal tissues is adjacent to ICA, so extended resection will be more challenging for surgeons. Therefore, the salvage surgery achieved better survival results in rT1-T2 patients and partial selected rT3 patients, and the efficacy of salvage surgeries on rT4 was significantly different in each study.

Although the prognostic factors influencing outcome have been studied, conclusive data on the survival benefits are still lacking. RCTs to compare surgery alone and surgery with adjuvant therapy are needed in patients with positive margin status. As a result of the importance of margin status, the goal of nasopharyngectomy is to obtain a microscopically negative margin (37). For patients with positive surgical margin status, further resection should be performed as soon as possible if the operation is feasible. In recent years, there is increasing evidence that histologically normal margins may have underlying genetic mutations that lead to negative margin results (38, 39). This has motived researchers to search for novel molecular markers to accurately predict local tumor recurrence after surgery (40).

Our study has several limitations. First, patients undergoing surgery alone are highly selected, with a greater chance of negative margins and better survival outcomes, which may increase the bias. The use of an open approach in patients with extensive invasion of the skull base or intracranial area also increase the potential bias. Second, based on the current research, it is not possible to recoend the optimal total dose, fractionated dose and fractionated method of exposure, which may lead to different results in different centers and thus lead to bias. Third, some raw survival data could not be obtained directly from the studies, and data obtained through statistical methods may not be accurate.

## Conclusion

The meta-analysis indicated that recurrent NPC patients can obtain survival benefits from salvage surgery. Accurately assessing the rT stage of the tumor and choosing the appropriate surgical method is of great significance to the success of the surgery. Although the prognostic factors influencing outcome have been studied, conclusive data on the survival benefits are still lacking, and RCTs to compare surgery alone and postoperative adjuvant therapy are necessary in patients with positive margin status after salvage surgery.

## Data Availability Statement

The original contributions presented in the study are included in the article/**Supplementary Material**. Further inquiries can be directed to the corresponding authors.

## Author contributions

YF participated in the retrieval and analysis of the article and drafted the manuscript. YF and ZD participated in the screening and data statistics of the article. RY, XZ, HY, CQ, CC, and SF reviewed and edited the manuscript. YP and HS participated in article screening and management, and reviewed, and edited the manuscript. All authors contributed to the article and approved the submitted version.

## Funding

This work was supported by National Nature Science Foundation of China #81600801 (HS).

## Conflict of Interest

The authors declare that the research was conducted in the absence of any commercial or financial relationships that could be construed as a potential conflict of interest.

## Publisher’s Note

All claims expressed in this article are solely those of the authors and do not necessarily represent those of their affiliated organizations, or those of the publisher, the editors and the reviewers. Any product that may be evaluated in this article, or claim that may be made by its manufacturer, is not guaranteed or endorsed by the publisher.
